# Rates of morphological evolution, asymmetry and morphological integration of shell shape in scallops

**DOI:** 10.1186/s12862-017-1098-5

**Published:** 2017-12-08

**Authors:** Emma Sherratt, Jeanne M. Serb, Dean C. Adams

**Affiliations:** 1 Department of Genetics and Evolution, School of Biological Sciences, The University of Adelaide, Adelaide, South Australia 5005 Australia; 2 Department of Ecology, Evolution, and Organismal Biology, Iowa State University, Ames, IA 50011 USA; 3 Department of Statistics, Iowa State University, Ames, IA 50011 USA

**Keywords:** Geometric morphometrics, Bivalve, Mollusc, Pectinidae, Morphological integration, Tempo and mode

## Abstract

**Background:**

Rates of morphological evolution vary across different taxonomic groups, and this has been proposed as one of the main drivers for the great diversity of organisms on Earth. Of the extrinsic factors pertaining to this variation, ecological hypotheses feature prominently in observed differences in phenotypic evolutionary rates across lineages. But complex organisms are inherently modular, comprising distinct body parts that can be differentially affected by external selective pressures. Thus, the evolution of trait covariation and integration in modular systems may also play a prominent role in shaping patterns of phenotypic diversity. Here we investigate the role ecological diversity plays in morphological integration, and the tempo of shell shape evolution and of directional asymmetry in bivalved scallops.

**Results:**

Overall, the shape of both valves and the magnitude of asymmetry of the whole shell (difference in shape between valves) are traits that are evolving fast in ecomorphs under strong selective pressures (gliders, recessers and nestling), compared to low rates observed in other ecomorphs (byssal-attaching, free-living and cementing). Given that different parts of an organism can be under different selective pressures from the environment, we also examined the degree of evolutionary integration between the valves as it relates to ecological shifts. We find that evolutionary morphological integration is consistent and surprisingly high across species, indicating that while the left and right valves of a scallop shell are diversifying in accordance with ecomorphology, they are doing so in a concerted fashion.

**Conclusions:**

Our study on scallops adds another strong piece of evidence that ecological shifts play an important role in the tempo and mode of morphological evolution. Strong selective pressures from the environment, inferred from the repeated evolution of distinct ecomorphs, have influenced the rate of morphological evolution in valve shape and the magnitude of asymmetry between valves. Our observation that morphological integration of the valves making up the shell is consistently strong suggests tight developmental pathways are responsible for the concerted evolution of these structures while environmental pressures are driving whole shell shape. Finally, our study shows that directional asymmetry in shell shape among species is an important aspect of scallop macroevolution.

**Electronic supplementary material:**

The online version of this article (10.1186/s12862-017-1098-5) contains supplementary material, which is available to authorized users.

## Background

It has long been observed that rates of morphological evolution vary across different taxonomic groups [1–3] and variation in rates of evolution has been proposed as one of the main drivers for the great diversity of organisms on Earth (e.g., [4]). A central aim in evolutionary biology is therefore to document the observed variability in the tempo of evolution in groups of closely related species and identify extrinsic and intrinsic factors pertaining to this variability. Fortunately, this is now possible because of the accumulation of large quantitative phenotypic datasets and advances in phylogenetic comparative methods [5–14]. Historically, variability in the tempo of morphological diversification across clades has been investigated with respect to phyletic factors such as lineage diversification, species richness or clade age [15–19]. While these are likely correlates, aspects of an organism’s biology, ecology and development are arguably the underlying factors driving evolutionary changes in morphology.

Ecological hypotheses feature prominently in observed differences in phenotypic evolutionary rates across lineages. Transitioning from one ecological niche to another can promote changes in the tempo of morphological evolution by either limiting morphological diversification or substantially accumulating change (e.g., [20, 21–24]). For example, rock-dwelling and arboreal lizard species display reduced rates of morphological evolution compared to their terrestrial counterparts [22], while coral reef habit is correlated with increased rates of phenotypic evolution in fish [20, 23]. Indeed, ecological opportunity is a primary factor implicated in regulating the tempo of morphological diversification in adaptive radiations [25–28]. In a similar manner, elevated rates of phenotypic change are typically found in organisms occupying disturbed or novel environments (e.g., [29]) or facing high predation pressures [30, 31]. Together these studies demonstrate that the habitat use and an organism’s environment affect the rate at which morphological diversity accumulates.

In some cases, external selection pressures can act differentially on separate traits of the same individual, resulting in variability in the rates of phenotypic evolution within organisms (e.g., [12, 32, 33–36]). For example, traits under sexual selection versus those under natural selection experience distinct selective regimes, which can result in different rates of evolutionary changes among traits [32]. Furthermore, traits that are under strong selective pressures resulting from biomechanical constraints [36], or those that perform several functions and face tradeoffs [37], may exhibit lower rates of morphological evolution than other traits not involved in such behaviours [36, 37]. Under such circumstances, differential selection across traits may cause an evolutionary decoupling, where some sets of traits covary tightly with one another but are relatively independent from other sets of traits. When viewed across a phylogeny, this pattern is known as evolutionary morphological integration [38]. Integration is expected to decrease when each trait is under different, possibly antagonistic, selection pressures [39], such as in the evolutionary decoupling of mammalian fore- and hind-limbs as a result of life history changes [40] or different locomotory behaviour [41]. These examples also demonstrate how an animal’s ecology influences evolutionary morphological integration. Therefore, integration and the tempo of morphological change are expected to be tightly linked in macroevolution, and explicitly examining the degree of integration between different traits is necessary when estimating the tempo of morphological evolution of sets of traits [13, 36, 42].

Morphological evolution of bivalved scallops (Pectinidae) is strongly influenced by ecological niche shifts [43–46], and thus they present an attractive system with which to study the role ecological diversity on the dynamics of phenotypic evolution. Scallops exhibit substantial diversity of shell morphologies related to the ecology (“life habit” sensu [47]) of the animal: adult scallops can be broadly organised into six ecomorphs that vary in their level of mobility (cementing, nestling, byssal-attaching, recessing, free-living, and gliding (long-distance swimming): [44, 47]). Further, the mode of shell shape evolution in scallops differs among ecomorphs. For example, recessing scallops, those that bury themselves under sand, have evolved by directional evolution resulting in the most derived species displaying the most pronounced concave left valve shape [45]. Gliding scallops, those with high-performance swimming ability (described in [48]), have convergently evolved a disc-like, flattened valve shape [43, 46]; while byssal-attaching and free-living scallops are morphologically similar within ecomorph but display substantial evolutionary plasticity [45]. Given these different modes of evolution, it is likely that the tempo of morphological evolution may differ among ecomorphs as well, but to date this hypothesis has yet to be examined.

The scallops are also an attractive system with which to examine evolutionary morphological integration. The bivalve shell is a simple modular system comprised of two valves attached with a hinge on the dorsal side. This two-valved arrangement poses an interesting challenge to studying morphological evolution, as bivalved animals can have both object symmetry (anterior-posterior symmetry within a valve) as well as matching symmetry (degree of similarity in shape and size between the left and right valves) [49]. Note that object symmetry, relating to the anterior-posterior axis along a single valve, is not considered here, and thus asymmetry discussed herein refers to matching symmetry of valves. Most bivalves are orientated such that the hinge is perpendicular to the surface of a substrate. Because the two valves experience similar environmental conditions, there is a strong expectation of symmetry between the left and right valves [47, 50]. However, scallops are unlike most bivalves as they live on the surface of a substrate (epifaunal) with the right valve (lower valve) usually in contact with the substrate, while the left valve (upper valve) is more often exposed ([51], but see [52]). This orientation predicts that the two valves will experience different selective pressures resulting in asymmetry between the two valves (discussed in [47]). Here we refer to this as directional asymmetry (DA), the consistent difference between a pair of morphological structures [53, 54]. Importantly, DA in scallops has never been quantified at a macroevolutionary scale, and the degree to which the two valves are evolving in concert (morphological integration) has yet to be tested.

Here we investigate the role ecological diversity plays in morphological integration and tempo of shell shape evolution and directional asymmetry in scallops. We estimate the magnitude of morphological integration in scallop shell shape across species, and examine how the strength varies among ecomorphs to understand how the complex shell is evolving in response to changing ecological demands. Then we estimate the tempo of evolution in three traits: the left valve shape; the right valve shape; and the difference in shape between valves (amount of asymmetry), which is a trait representing the whole shell. With these estimates, we test whether the tempo of morphological change differs among ecomorphs, and whether there are within-shell differences in rates (comparing left versus right valves). We predict substantial differences of morphological integration and tempo among ecomorphs because of the differing functional constraints imposed by the life habit, the degree of exposure each valve contends with, and the different roles of the left and right valves. Further, we expect particularly high rates of morphological change and stronger integration in gliding and recessing species because these ecomorphs have been previously identified to be under strong natural selection [43–46]. We characterise shell shape (left and right valves) with geometric morphometrics for 86 species across the six ecomorphs, and for the remainder of the paper, we follow the malacological literature and use the terms ‘equivalve’ (similar) and ‘inequivalve’ (dissimilar) to describe the degree of asymmetry between the left and right valves [55]. Shape data are analysed in a phylogenetic framework using recent statistical advances for multidimensional traits that permits morphological integration to be examined in an evolutionary context [56] and net phenotypic evolutionary rates to be calculated between traits of the same individuals and between groups of species [11, 13]. Implications of our findings are discussed in terms of how rates of evolution and the evolution of morphological integration, impact macroevolutionary trends of phenotypic diversity.

## Methods

### Samples and morphometric analyses

We sampled shells from 1081 adult individuals for 123 species and spanning the range of ecomorphs (museum collections listed in Additional file 1: Table S1 and Acknowledgments). However, because not all shells are preserved with both valves intact, nor are all species included in the most current molecular phylogenetic hypothesis [45], the total number of shells available to study was 669 individuals from 86 species (2 cementing, 1 nestling, 48 byssal-attaching, 11 recessing, 18 free-living, and 6 gliding species). These proportions of ecomorph categories in our taxonomic sample are similar to their representation across extant species the family (in square brackets): cementing = 2% [1.9%]; nestling = 1% [0.75%]; byssal-attaching = 57% [66%]; free-living = 20% [16.3%]; recessing = 12% [12.1%]; gliding = 6% [3%] [44].

**Fig. 1:**
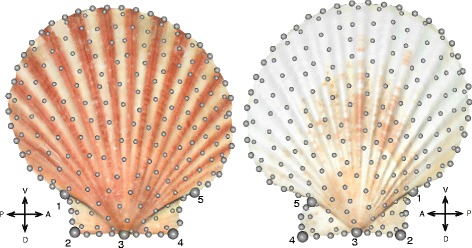
Three-dimensional surface scans of the left and right valves of a representative scallop, with the landmarks and semilandmarks shown. Five landmarks are numbered and represented by large dots and the semilandmarks are shown as small dots. Landmark 1: ventro-posterior auricle, 2: dorsoposterior auricle, 3: umbo, 4: dorsoanterior auricle, 5: ventroanterior auricle. In life, the left valve (‘upper’) is usually oriented upwards, with the right valve (‘lower’) against the substrate, hence the visible colour differences shown here

Landmark data collection follows that of previous studies [43, 45, 46] and is presented in more detail in Sherratt et al. [45]. In summary, we obtained three-dimensional (3D) surfaces representing the left and right valves using a NextEngine 3D scanner (Next Engine Inc., Santa Monica, CA). Next, valve shape was characterised using landmark-based geometric morphometrics [57–59]. We used a combination of fixed landmarks representing homologous points and semilandmarks, points on curves and surfaces [58, 60]. On each 3D surface, we placed 5 landmarks around the auricles and umbo (Fig. 1). Scallop left and right valves have matching symmetry along the line of contact between the closed valves (i.e., commissural plane; [61]), thus the right valves were digitised so that the landmark points are a reflection along the dorsal/ventral axis. Then, 197 equally-spaced landmarks were digitised on the valve surface to characterise the boundary contours of the valve and auricles as well as the valve curvature in the z dimension. Each valve was measured twice to account for measurement error. The landmark data from the left and right valves were treated as separate structures and were thus aligned separately using a generalised Procrustes superimposition [62], where the semilandmarks were permitted to slide along their tangent directions [60] in order to minimise Procrustes distance between specimens. Morphometric analyses were performed in R v.3.3.1 [63] using the *geomorph* library v.3.0.4 [64].

### Comparative analyses

Because species are not independent of one another, all statistical analyses evaluating our predictions were conducted using a phylogenetic comparative framework. We used a time-calibrated molecular phylogeny [45] that was pruned to only include the 86 species in this study. To examine morphological integration and characterise the patterns of shape covariation between the left and right valves across all species, we used two-block partial least squares (PLS) analysis [65] and evaluated the statistical significance in a phylogenetic context [56]. The strength of covariation between the valves was calculated as the correlation coefficient (*r*_PLS_) of the first PLS axis of each valve. The *r*_PLS_ was calculated for all 86 species using species mean shapes, as well as for each ecomorph (excluding the single nestling species). Each test was statically assessed by a permutation procedure (1000 permutations each). To test whether the strength of integration differs between ecomorphs, we compared their effect sizes (*Z*-scores), which provides a standardised measure of the strength of integration in each dataset for formal quantitative comparisons [66]. To visualise the pattern of morphological integration, we performed a two-block PLS analysis of all 669 specimens and plotted the first PLS axes of the left valve against that of the right. The shape changes along these axes was visualised using a thin-plate spline method to warp a 3D surface mesh of each valve to the mean shape and from there to the minima and maxima of the axes (e.g., [67, 68]).

The magnitude of asymmetry was estimated as the absolute difference between left and right valve shape, measured as Procrustes distance. To do this, we performed a second Procrustes superimposition of the left and right valves together (where right valves were mirrored along the anterior-posterior axis to match the left valve), again allowing semilandmarks to slide, and calculated the Procrustes distance between left and right valves for all 669 specimens. To test whether the amount of individual asymmetry differed across ecomorphs while accounting for phylogeny, we used a phylogenetic generalised least squares analysis for multidimensional data (PGLS 45, [69]) implemented in *geomorph*. The significance of the model was evaluated via permutation (1000 iterations).

The tempo of morphological evolution was estimated using the net evolutionary rate parameter under a Brownian motion model of evolution (σ^2^) for valve shape among species on the phylogeny. We addressed four hypotheses to examine differences in evolutionary rate between valves and among ecomorphs: 1) the rate of DA evolution will differ among ecomorphs 2) the net rate of valve shape evolution will differ between left and right valves across all species 3) the net rate of valve shape evolution will differ among ecomorphs for the a) left valve and b) right valve 4) the rate of evolution will differ between valves within each ecomorph. To do this, we used an approach for multidimensional shape data which obtains estimates of the net evolutionary rate of change in the multidimensional morphospace after phylogenetic transformation [11, 13]. For each hypothesis, evolutionary rates for each ecomorph or trait were obtained, and from them test statistics calculated (a ratio of maximum to minimum evolutionary rates: see Adams 2014b; Denton and Adams 2015). Statistical significance of the observed test measures was then evaluated via phylogenetic simulation, in which tips data were obtained under the null hypothesis of a single Brownian motion process for all species on the phylogeny [11, 13]. For all evolutionary rate estimations, 95% confidence intervals were estimated by bootstrapping the individuals used to calculate the species means (1000 iterations). Phylogenetic analyses were performed using the *phytools* library v.0.5–38 [70], the *APE* library v.3.5 [71], and the *geomorph* library v.3.0.4 [64].

## Results

**Fig. 2:**
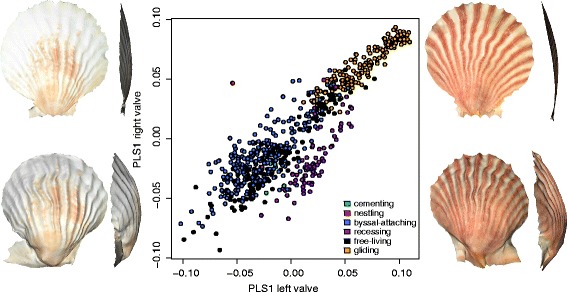
Morphological integration as described by a two-block PLS analysis of the left and right valves of all 669 specimens. Most equivalve specimens are distributed along the central axis of the scatter, and most inequivalve specimens would lie furthest from the central axis. Thin-plate spline warped surface scans depicting valve shape at the minima and maxima of PLS1 for the left valve and PLS1 for the right valve are shown, to the right and left of the biplot respecively

The overall strength of evolutionary integration between the left and right valve across all species was moderately high (*r*_PLS-all_ = 0.80, *P* < 0.001). When examined by ecomorph, we found the more motile ecomorphs displayed very high levels of integration (*r*_PLS-free_ = 0.95, *r*_PLS-glide_ = 0.94), while species in the sessile ecomorphs exhibited slightly lower levels of integration (*r*_PLS-byssal_ = 0.88, *r*_PLS-recess_ = 0.88, all *P* < 0.05; cementing and nestling excluded due to sample size). However, the strength of morphological integration (*r*_PLS_) did not differ significantly among these ecomorphs (effect sizes, *Z*: byssal = 6.5, recess = 2.6, free = 4.4, glide = 1.5, *P*-values in Additional file 1: Table S2), although there was a trend for gliders to be marginally more integrated than the byssal-attaching and free-living species (both *P* = 0.06). The pattern of integration between left and right valves among all 669 specimens is shown in Fig. 2. Here, most specimens fell along a single trajectory of shape covariation, indicating concerted evolutionary change in both valves’ morphology among species. The shape changes associated with the first PLS axes of each valve (Fig. 2) described similar changes, from a highly convex valve to a flattened valve. The gliding species lied in the region where both valves are very flat, while the byssal-attaching and free-living occupied the region where both valves were convex. The recessing species were a clear exception to this pattern, being offset from the rest – where their right valve was convex and their left valve was flattened.

**Fig. 3:**
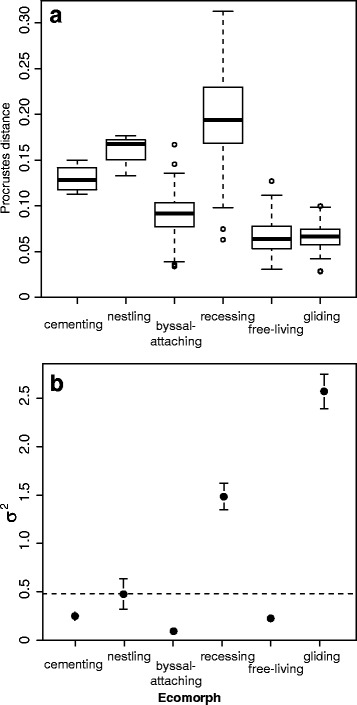
Asymmetry in scallop shells among ecomorphs. **a**: degree of asymmetry, measured as absolute Procrustes distance between left-right valve shapes for all specimens, shown by ecomorphs. Procrustes distance 0, equivalve; more than 0, inequivalve. **b**: the rate of asymmetry (DA) evolution (σ^2^) among ecomorphs. Abscissa values are ×10^−4^. Dashed line represents the overall evolutionary rate of asymmetry across all species. Confidence intervals calculated via bootstrapping specimens in species means estimations

The magnitude of shell asymmetry was highly variable among species and clearly differed within and between ecomorphs (Fig. 3a, Additional file 1: Figure S1, Table 1A). Recessing species displayed the highest mean asymmetry, as well as the largest variance in asymmetry, while gliding species and free-living species exhibited the lowest mean asymmetry. However, a PGLS of the species’ means of asymmetry was not significant between ecomorphs (*F* = 1.19, *P* = 0.805), suggesting that differences in asymmetry were not unexpected when shared evolutionary history was taken into consideration. The rate of DA evolution was quite high for recessing species and gliding species, and low for all other ecomorphs (Fig. 3b), but overall there was no significant difference among ecomorphs (σ^2^_6 habit ratio_ = 28.2, *P* = 0.573) despite some significant pairwise differences (Table 1A).

**Table 1:** Pairwise *P*-values for comparisons of morphological rate estimates for degree of asymmetry (A), left valves (B) and right valves (C) among ecomorphs

	cementing	nestling	byssal-attaching	recessing	free-living
A)					
nestling	0.758				
byssal-attaching	0.744	0.631			
recessing	0.717	0.877	**0.006**		
free-living	0.968	0.82	0.201	0.133	
gliding	0.201	0.532	**0.002**	0.914	**0.037**
B)					
nestling	0.385				
byssal-attaching	0.327	0.787			
recessing	0.036	**0.004**	**0.001**		
free-living	0.51	0.106	**0.001**	**0.011**	
gliding	0.1	**0.017**	**0.001**	0.573	0.092
C)					
nestling	0.099				
byssal-attaching	**0.009**	**0.001**			
recessing	0.141	0.431	**0.001**		
free-living	0.091	**0.002**	**0.016**	**0.001**	
gliding	0.284	0.332	**0.001**	0.706	**0.001**

**Fig. 4:**
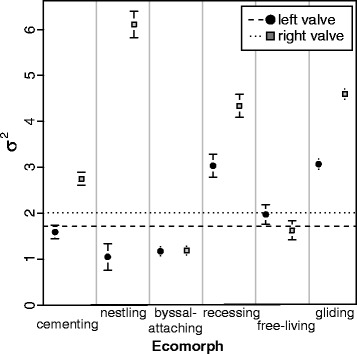
Phylogenetic evolutionary rate (σ^2^) of shape evolution among ecomorphs for the left (circle) and right (square) valves, with their 95% CIs. Abscissa values are ×10^−7^. Dashed and dotted lines represent the overall evolutionary rate for the left and right valves respectively. Pairwise comparisons between ecomorphs by valve are presented in Table 1. Confidence intervals were calculated via bootstrapping specimens in species-means estimations

*P*-values significant at the 5% level are given in bold. See Fig. 3b and Fig. 4 for corresponding rate estimates

Comparing across all species, the left and right valves differed significantly in overall rates of shape evolution (σ^2^_R/L ratio_ = 1.17, *P* < 0.001), with a distinctly lower rate for left valves than for right valves (σ^2^_left_ = 1.72 × 10^−7^, σ^2^_right_ = 2.01 × 10^−7^) (Fig. 4, abscissa). This indicated that the right valve, which is the one usually in contact with the substrate, was more evolutionary labile in its morphology over macroevolutionary time.

Comparing across ecomorphs, the rates for left-valve shape evolution were significantly different (Fig. 4, circles. σ^2^_6 habit ratio_ = 2.92, *P* = 0.028, Table 1B). Recessing species and gliding species showed the highest rates, more than double that of the lowest rates, seen in byssal-attaching and nestling species. Most pairwise rate differences among ecomorphs were not significantly different. Notably, the byssal-attaching species were evolving significantly slower that the recessing, free-living and gliding species.

The right valve displayed a more prominent pattern of variability in evolutionary rates, where there were substantial and significant differences among ecomorphs (Fig. 4, squares. σ^2^_6 habit ratio_ = 5.16, *P* = 0.001, Table 1C). Here, the nestling species displayed the highest rate, followed jointly by the recessers and gliders, while the byssal and free-living species exhibited the lowest rates of shape evolution. Biologically, this difference in evolutionary rates corresponds to differences in natural history, as in scallops the right valve is predominantly in contact with the substrate. For instance, in recessing species that excavate and then settle the right valve into a depression in sand, the right valve has evolved to be substantially more convex compared to that of the byssal-attachers. Conversely, the gliding species have evolved a much flatter right valve, relative to those of free-living and byssal-attaching species. The byssal-attaching species are evolving significantly slower than all the other life-habits.

Focussing within each ecomorph, the difference in evolutionary rates between left and right valves differed among ecomorphs in terms of statistical significance, but generally, the right valve shape evolved faster than the left (except the free-living ecomorph, Fig. 4). In the single nestling species, *Pedum spondyloideum,* the rate of evolution in the right valve was almost six times higher than the left (σ^2^_R/L ratio_ = 5.82). For the two cementing species, the rate of evolution in the right was higher than the left (σ^2^_R/L ratio_ = 1.73). Gliding and recessing species also showed much higher rates of evolution in the right valve (gliding σ^2^_R/L ratio_ = 1.96, *P* = 0.031; recessing σ^2^_R/L ratio_ = 1.67, *P* = 0.014). In contrast, the left valve of free-living species displayed higher rates than the right valve, but these were not significantly different (σ^2^_R/L ratio_ = 0.94, *P* = 0.594). Byssal-attaching species had low rates for both valves and these rates were not significantly different (σ^2^_R/L ratio_ = 1.00, *P* = 0.965).

## Discussion

Bivalved scallops have evolved a diverse array of shell shapes, by a variety of evolutionary modes (i.e., divergence, convergence and directional evolution) in response to macroevolutionary transitions between ecomorphs [43–46]. This study adds to a growing body of literature on macroevolution of scallops by demonstrating that these ecomorphs also differ substantially in the tempo of shell shape evolution (both valves) and in the magnitude of DA between the left and right valves, but not in the strength of morphological integration.

At the species level, bivalved scallop shells are evolving as an integrated unit. Our results suggest that left and right valves of all sampled species are evolving in concert, displaying moderate to high degrees of shape covariation (integration), whose levels are comparable across ecomorphs. To the best of our knowledge, the strength of morphological integration between valves of scallops, or indeed any other bivalved molluscs, has not been previously examined using geometric morphometric methods to quantify shape covariation. Our study complements the theoretical discussion of phenotypic integration, which has been presented as an important facet of bivalve evolution (e.g. [72, 73, 74]). Morphological integration is a central aspect of macroevolution, because the degree of correlated evolution among structures is expected to influence the evolvability of a clade [75–77]. The two valves of bivalve molluscs are thought to have developed in from a single-valved ancestral condition [78, 79] where the shell field elongated dorso-ventrally and subsequently folded along the mid-dorsal region [47, 80]. This would suggest that the two valves are closely associated during development and share the same gene regulatory pathways [81]. The strength of covariation between parts of articulated morphological structures would be much lower than what we observed here if the two valves are evolving as separate ‘modules’, for example in mammalian limbs [40, 41] and mantis shrimp claws [82, 83]. We propose that high evolutionary integration in scallop shells across all species is the result of a functional constraint for the hinge elements and shape of the commissural margin between left and right valves to interlock.

Nevertheless, in spite of the strong phenotypic integration found between valves, we found that the differences in the shape between the left and right valves − the magnitude of DA − varied substantially among species of scallops and relates strongly to ecomorph type, and that this variation in asymmetry followed a continuum across the life habits displayed by the various ecomorphs. For example, the gliding species, along with free-living and byssal-attaching species, displayed the lowest levels of asymmetry and thus were the most equivalved in their shell shape, whilst more sedentary taxa found in the recessing, nestling and cementing ecomorphs displayed much greater asymmetry in shell shape (inequivalved). This pattern may thus be interpreted as a reflection of the way in which the different ecomorphs locomote, as species displaying a more active lifestyle (e.g., gliding) must have a higher degree of symmetry between the right and left valves to reduce hydrodynamic drag (e.g., [43, 84]).

With respect to the pace of evolutionary change, the tempo of the three shell traits (shell asymmetry, left and right valve shape) along the branches of the phylogeny all present a similar pattern among ecomorphs: rates are high in gliding, recessing and nestling species, while comparatively low for the other ecomorphs. This is particularly conspicuous in the shape of the right valve and thus overall shell asymmetry (Figs. 3b & 4). The right valve is often in contact with the substrate in bivalve species that are in a non-vertical position and evolves different shapes suited to the life habit. For example, the single nestling species, *Pedum spondyloideum*, is an obligate commensal, where the scallop settles on and then becomes encased as the living scleractinian corals grows around it [85]. Within this chamber, the right valve becomes immobile, while the left valve can move during adduction of the valves. As a result, the right valve is modified during growth to maximise shell gape by displacing the hinge line and the migration of the byssal notch [86]. Higher rates of asymmetry and shape evolution in nestling species in this study are expected for the same environmental reasons. This agrees with what has been shown in other taxa: great morphological variability in shell shape has been described in similar crevice-dwelling species of pterioid bivalves [87], which suggests that the variability is inherent property of this specialised life habit. Thus, our observed high rates of shape change in the right valves of species of these three scallop ecomorphs is predicted to be due to natural selection and their respective natural histories.

AP Møller and A Pomiankowski [88] proposed that estimating the degree of trait fluctuating asymmetry (FA), the small asymmetrical differences due to developmental perturbations, would be a good proxy for estimating the pace of evolutionary change. FA reflects the ability of an individual to cope with stress, and traits under strong directional selection, (thus experiencing elevated rates of phenotypic evolution, Lande 1976) would become more susceptible to the influence of stress. They suggested that phenotypic traits from periods of rapid evolutionary change should display high FA and morphological variability, while traits from periods of slow evolutionary change should display low FA and variability. Do our results from scallops support this prediction? We did not measure FA here, but we did measure an evolutionary outcome, directional asymmetry (the difference between the left and right valves). The most inequivalve (highly asymmetrical) ecomorph, where the left value is distinctly flattened and the right valve exhibits a great deal of convexity variation across species, is the recessing scallops. A previously reported directional trend in left valve shape [45] appears to coincide with increasing convexity of the right valve and explains the increasing shell asymmetry we observe in this ecomorph. This trend is a consequence of a macroevolutionary transition from an epifaunal to semi-infaunal existence (see [45] for discussion). The recessing scallops showed high variation in asymmetry across species (Fig. 3a), and elevated rates of evolutionary change (Fig. 4), thus supporting the hypothesis of AP Møller and A Pomiankowski [88]. At the other end of the asymmetry spectrum are the gliding scallops, the most equivalve ecomorph, because the highly-symmetrical left and right valves jointly function as an airfoil during the swimming behaviour [84]. The high rates of shape evolution of both valves, but particularly the left, in all gliding species is probably associated with natural selection for a convergent gliding morphology (discussed in [43]). However, this ecomorph displayed lower variation in asymmetry across species (Fig. 3a), and thus the hypothesis of AP Møller and A Pomiankowski [88] is not supported in this instance. The tendency for scallop shells to evolve back and forth along the asymmetry spectrum, displaying varying degrees of directional asymmetry, while retaining strongly integrated valves, is clearly an important facet of phenotypic evolution in this clade that deserves further investigation.

Finally, placing our results here in the context of a growing body of macroevolutionary work on scallops [43–46] provides several general insights. First, the neutral model of phenotypic evolution provides a framework for the understanding of rates of morphological evolution: unexpectedly low rates result from stabilising selection (e.g., [36]), while high rates of evolution imply natural selection including directional evolution [89]. Scallop macroevolution appears to meet these expectations: there is a baseline low rate of change in shell asymmetry and valve shape among species of the byssal-attaching and free-living ecomorphs (the ancestrally-reconstructed ecomorph state for scallops; [44]), and only along the branches towards the gliding, recessing and nestling species do we see a marked increase in rates. Second, gliding, recessing and cementing ecomorphs have evolved independently multiple times [44, 46] and with morphologically similar outcomes (valve shape and shell asymmetry), supporting the predictability of evolution (e.g., [90, 91]). Finally, the evolution of directional asymmetry in scallop shells is shown here to be a fundamental component of scallop macroevolution and is offered as a novel system in which to study the concept of adaptive asymmetry, where fluctuating asymmetry between left and right paired structures provides a selective advantage and becomes directional asymmetry at a macroevolutionary scale [92].

## Conclusions

Our study on scallops adds another strong piece of evidence that ecological shifts play an important role in the tempo and mode of morphological evolution. The results presented here suggest that strong selective pressures from the environment, inferred from the repeated evolution of distinct ecomorphs, have influenced the rate of morphological evolution in scallop valve shape and the magnitude of asymmetry between left and right valves. We observed strong morphological integration between the two valves comprising the shell, and this is consistent across all ecomorphs, which suggests there are distinct, evolutionarily conserved developmental pathways responsible for the concerted evolution of these structures even though environmental pressures are driving whole shell shape. In conclusion, an interplay and morphological integration and directional asymmetry in shell shape among species have played an important role in scallop macroevolution.

## Additional file

Additional file 1: Figure S1. Variation in matching asymmetry in scallop shells across the 86 species, colored by life habit. **Table S1.** Morphometric data of left and right valves were available for 86 species comprising six life habits. **Table S2.** Significance (*P*-values) for pairwise comparisons of effect sizes, Z scores, from partial least squares analysis. (DOCX 220 kb)

## Abbreviations

DA: Directional asymmetry
FA: Fluctuating asymmetry
PLS: Two-block partial least squares
