# Supplementary material for: Rates of morphological evolution, asymmetry and morphological integration of shell shape in scallops
Source: BMC Evol Biol. 2017 Dec 8;17:248. doi: 10.1186/s12862-017-1098-5 (PMC5721563; doi:10.1186/s12862-017-1098-5)
Supplement: Additional file 1: Figure S1. — Variation in matching asymmetry in scallop shells across the 86 species, colored by life habit. Table S1. Morphometric data of left and right valves were available for 86 species comprising six life habits. Table S2. Significance (P-values) for pairwise comparisons of effect sizes, Z scores, from partial least squares analysis. (DOCX 220 kb) [file 12862_2017_1098_MOESM1_ESM.docx]

**Supporting Information for**

Rates of morphological evolution, asymmetry and morphological integration of shell shape in scallops (Bivalvia: Pectinidae)

**Authors:**

E. Sherratt, J.M. Serb and D.C. Adams

(1 supplementary figure and 2 supplementary tables)

**Figure S1** Variation in matching asymmetry in scallop shells across the 86 species, colored by life habit (green = cementing, red = nestling, blue = byssal-attaching, purple = recessing, black = free-living, orange = gliding).

**
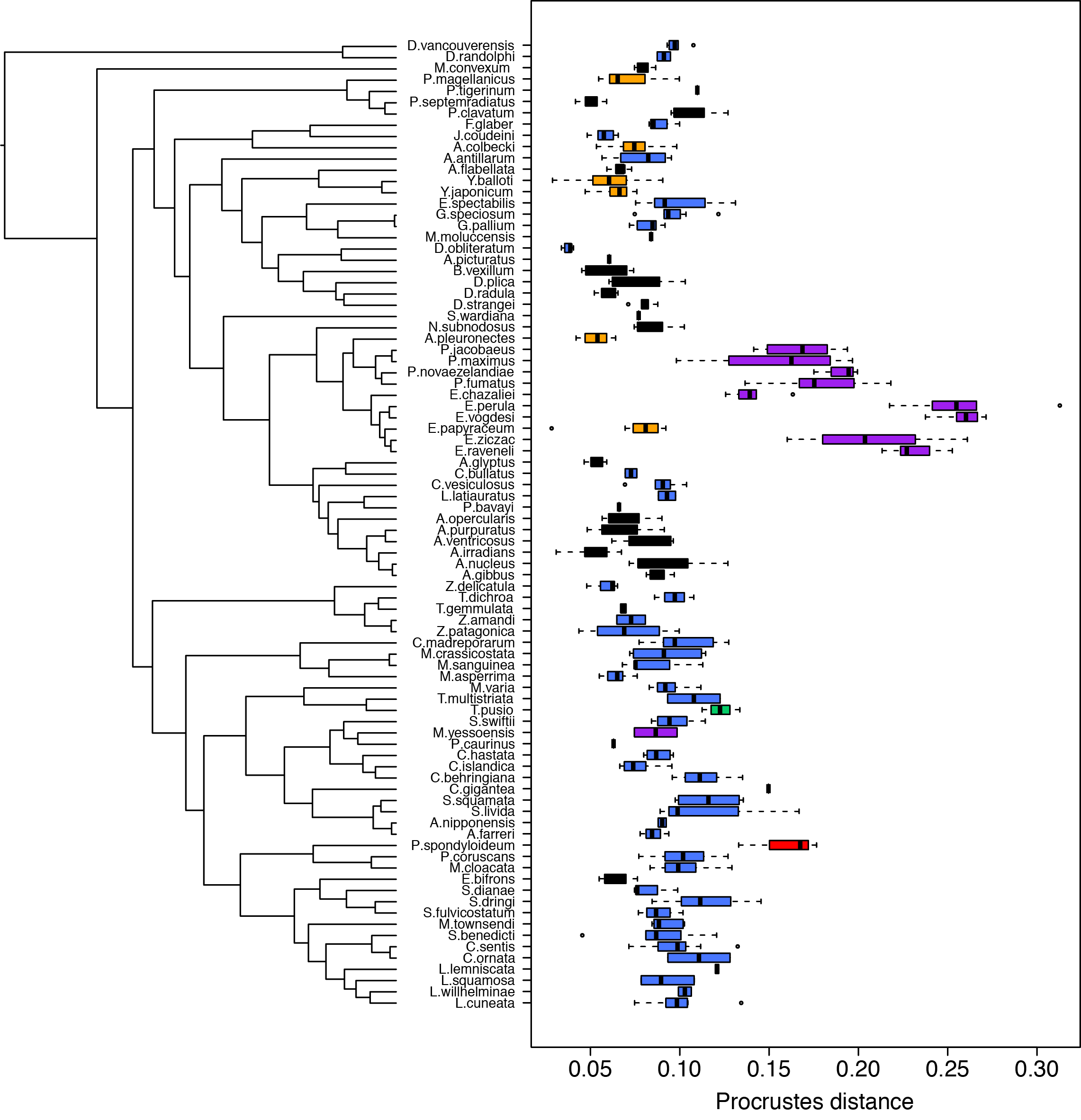
**

**Table S1** Morphometric data of left and right valves were available for 86 species comprising six life habits. The ID corresponds to the label of Figure S1. Number of specimens used (No. spec.) to calculate the average for each species were taken from museum collections, summarized here using the official museum acronyms. Free, free-living. Byssal, byssal-attaching.

| **Species** | **ID** | **Habit** | **No. spec.** | **Museum** |
| --- | --- | --- | --- | --- |
| ***Adamussium colbecki*** | *A.colbecki* | gliding | 39 | USNM |
| ***Aequipecten glyptus*** | *A.glyptus* | free | 5 | FMNH |
| ***Aequipecten opercularis*** | *A.opercularis* | free | 7 | FMNH |
| ***Amusium pleuronectes*** | *A.pleuronectes* | gliding | 30 | USNM |
| ***Anguipecten picturatus*** | *A.picturatus* | free | 3 | MNHN |
| ***Annachlamys flabellata*** | *A.flabellata* | free | 5 | UF |
| ***Antillipecten antillarum*** | *A.antillarum* | byssal | 7 | UF; LACM; USNM; LACM |
| ***Argopecten gibbus*** | *A.gibbus* | free | 5 | LACM |
| ***Argopecten irradians*** | *A.irradians* | free | 28 | DMNH |
| ***Argopecten nucleus*** | *A.nucleus* | free | 10 | LACM |
| ***Argopecten purpuratus*** | *A.purpuratus* | free | 25 | UF |
| ***Argopecten ventricosus*** | *A.ventricosus* | free | 6 | LACM; CAS |
| ***Azumapecten farreri*** | *A.farreri* | byssal | 5 | LACM; BPBM |
| ***Azumapecten nipponensis*** | *A.nipponensis* | byssal | 2 | AMNH |
| ***Bractechlamys vexillum*** | *B.vexillum* | free | 10 | LACM |
| ***Caribachlamys ornata*** | *C.ornata* | byssal | 2 | BPBM |
| ***Caribachlamys sentis*** | *C.sentis* | byssal | 30 | UF |
| ***Chlamys behringiana*** | *C.behringiana* | byssal | 19 |  |
| ***Chlamys hastata*** | *C.hastata* | byssal | 13 | MCZ; BPBM |
| ***Chlamys islandica*** | *C.islandica* | byssal | 8 | YPM; LACM |
| ***Coralichlamys madreporarum*** | *C.madreporarum* | byssal | 9 | LACM; MNHN |
| ***Crassadoma gigantea*** | *C.gigantea* | cementing | 1 | NCSM |
| ***Cryptopecten bullatus*** | *C.bullatus* | byssal | 2 | MCZ; UF |
| ***Cryptopecten vesiculosus*** | *C.vesiculosus* | byssal | 5 | LACM |
| ***Decatopecten plica*** | *D.plica* | free | 10 | LACM |
| ***Decatopecten radula*** | *D.radula* | free | 10 | LACM; BPBM |
| ***Decatopecten strangei*** | *D.strangei* | free | 6 | LACM |
| ***Delectopecten randolphi*** | *D.randolphi* | byssal | 3 | MCZ |
| ***Delectopecten vancouverensis*** | *D.vancouverensis* | byssal | 7 | LACM |
| ***Dentamussium obliteratum*** | *D.obliteratum* | byssal | 5 | LACM; DMNH |
| ***Equichlamys bifrons*** | *E.bifrons* | free | 9 | LACM; DMNH; BPBM |
| ***Euvola chazaliei*** | *E.chazaliei* | recessing | 5 | NCSM; AMNH |
| ***Euvola papyraceum*** | *E.papyraceum* | gliding | 14 | FMNH |
| ***Euvola perula*** | *E.perula* | recessinging | 7 | UF |
| ***Euvola raveneli*** | *E.raveneli* | recessinging | 7 | LACM; YPM |
| ***Euvola vogdesi*** | *E.vogdesi* | recessing | 12 | LACM; USNM |
| ***Euvola ziczac*** | *E.ziczac* | recessing | 28 | FMNH; LACM |
| ***Excellichlamys spectabilis*** | *E.spectabilis* | byssal | 16 | LACM |
| ***Flexopecten glaber*** | *F.glaber* | byssal | 2 | MNHN; YPM |
| ***Gloripallium pallium*** | *G.pallium* | byssal | 9 | FMNH |
| ***Gloripallium speciosum*** | *G.speciosum* | byssal | 8 | LACM |
| ***Juxtamusium coudeini*** | *J.coudeini* | byssal | 6 | MNHN |
| ***Laevichlamys cuneata*** | *L.cuneata* | byssal | 8 | LACM; MNHN |
| ***Laevichlamys lemniscata*** | *L.lemniscata* | byssal | 5 | LCSM; MCZ; DMNH |
| ***Laevichlamys squamosa*** | *L.squamosa* | byssal | 8 | MNHN; CAS |
| ***Laevichlamys willhelminae*** | *L.willhelminae* | byssal | 4 | USNM |
| ***Leptopecten latiauratus*** | *L.latiauratus* | byssal | 5 | BPBM; NCSM; CAS |
| ***Mesopeplum convexum*** | *M.convexum* | free | 5 | MCZ; DMNH |
| ***Mimachlamys asperrima*** | *M.asperrima* | byssal | 7 | LACM |
| ***Mimachlamys cloacata*** | *M.cloacata* | byssal | 7 | MNHN; USNM |
| ***Mimachlamys crassicostata*** | *M.crassicostata* | byssal | 10 | FMNH |
| ***Mimachlamys sanguinea*** | *M.sanguinea* | byssal | 5 | MCZ; USNM; CAS |
| ***Mimachlamys townsendi*** | *M.townsendi* | byssal | 5 | USNM; AMNH |
| ***Mimachlamys varia*** | *M.varia* | byssal | 8 | FLMNH |
| ***Mirapecten moluccensis*** | *M.moluccensis* | byssal | 1 | MNHN |
| ***Mizuhopecten yessoensis*** | *M.yessoensis* | recessing | 5 | CAS; UF; AMNH |
| ***Nodipecten subnodosus*** | *N.subnodosus* | free | 4 | LACM; YPM |
| ***Palliolum tigerinum*** | *P.tigerinum* | byssal | 2 | CAS |
| ***Paraleptopecten bavayi*** | *P.bavayi* | byssal | 5 | DAMNH; UF |
| ***Pascahinnites coruscans*** | *P.coruscans* | byssal | 8 | FMNH USNM |
| ***Patinopecten caurinus*** | *P.caurinus* | recessing | 4 | CAS, MCZ; DMNH |
| ***Pecten fumatus*** | *P.fumatus* | recessing | 17 | LACM |
| ***Pecten jacobaeus*** | *P.jacobaeus* | recessing | 5 | NCSM; YPM |
| ***Pecten maximus*** | *P.maximus* | recessing | 6 | LACM |
| ***Pecten novaezelandiae*** | *P.novaezelandiae* | recessing | 5 | NCSM |
| ***Pedum spondyloideum*** | *P.spondyloideum* | nestling | 4 | MNHN; USNM;YPM |
| ***Placopecten magellanicus*** | *P.magellanicus* | gliding | 24 |  |
| ***Pseudamussium clavatum*** | *P.clavatum* | free | 5 | MCZ; AMNH |
| ***Pseudamussium septemradiatus*** | *P.septemradiatus* | free | 28 | USNM |
| ***Scaeochlamys livida*** | *S.livida* | byssal | 5 | FMNH; YPM; BPBM |
| ***Scaeochlamys squamata*** | *S.squamata* | byssal | 5 | MNHN; USNM |
| ***Semipallium dianae*** | *S.dianae* | byssal | 3 | MCZ; DMNH |
| ***Semipallium dringi*** | *S.dringi* | byssal | 24 | MCZ; MNHN |
| ***Semipallium fulvicostatum*** | *S.fulvicostatum* | byssal | 5 | MNHN; YPM; BPBM |
| ***Semipallium wardiana*** | *S.wardiana* | byssal | 1 | AMNH |
| ***Spathochlamys benedicti*** | *S.benedicti* | byssal | 5 | FMNH; DMNH |
| ***Swiftopecten swiftii*** | *S.swiftii* | byssal | 8 | DMNH; CAS |
| ***Talochlamys dichroa*** | *T.dichroa* | byssal | 3 | MNHN; DMNH |
| ***Talochlamys gemmulata*** | *T.gemmulata* | byssal | 5 | AMNH; BPBM; UF; MCZ |
| ***Talochlamys multistriata*** | *T.multistriata* | byssal | 4 | MNHN |
| ***Talochlamys pusio*** | *T.pusio* | cementing | 5 | BPBM; YPM; DMNH? |
| ***Ylistrum balloti*** | *Y.balloti* | gliding | 39 | WAMS; BALD ISL |
| ***Ylistrum japonicum*** | *Y.japonicum* | gliding | 36 | LACM; USNM |
| ***Zygochlamys amandi*** | *Z.amandi* | byssal | 3 | USNM |
| ***Zygochlamys delicatula*** | *Z.delicatula* | byssal | 5 | AMNH; MCZ |
| ***Zygochlamys patagonica*** | *Z.patagonica* | byssal | 14 | BPBM; YPM; LACM; UF |

**Table S2** Significance (P-values) for pairwise comparisons of effect sizes, Z scores, from partial least squares analysis.

|  | byssal | recess | free | glide |
| --- | --- | --- | --- | --- |
| byssal | - |  |  |  |
| recess | 0.1953 | - |  |  |
| free | 0.4041 | 0.1767 | - |  |
| glide | 0.0585 | 0.2578 | 0.0597 | - |
